# Impaired Global Longitudinal Strain Is Associated with Cardiovascular Events in Hodgkin Lymphoma Survivors

**DOI:** 10.3390/cancers14092329

**Published:** 2022-05-08

**Authors:** Elissa A. S. Polomski, Julius C. Heemelaar, Augustinus D. G. Krol, Marloes Louwerens, Saskia L. M. A. Beeres, Eduard R. Holman, J. Wouter Jukema, Martin J. Schalij, M. Louisa Antoni

**Affiliations:** 1 Department of Cardiology, Heart Lung Center, Leiden University Medical Center, 2333 ZA Leiden, The Netherlands; e.s.polomski@lumc.nl (E.A.S.P.); j.c.heemelaar@lumc.nl (J.C.H.); s.l.m.a.beeres@lumc.nl (S.L.M.A.B.); e.r.holman@lumc.nl (E.R.H.); j.w.jukema@lumc.nl (J.W.J.); m.j.schalij@lumc.nl (M.J.S.); 2 Department of Radiotherapy, Leiden University Medical Center, 2333 ZA Leiden, The Netherlands; a.d.g.krol@lumc.nl; 3 Department of Internal Medicine, Leiden University Medical Center, 2300 RC Leiden, The Netherlands; m.louwerens@lumc.nl

**Keywords:** cardio-oncology, Hodgkin lymphoma, echocardiography, global longitudinal strain, left ventricular dysfunction, left ventricular ejection fraction, thoracic radiotherapy

## Abstract

**Simple Summary:**

Radiotherapeutic treatment in classic Hodgkin lymphoma (CHL) survivors contributes to long-term survival but is associated with lifetime increased risk of cardiovascular events. Echocardiographic screening for left ventricular (LV) dysfunction usually assesses left ventricular ejection fraction (LVEF). Global longitudinal strain (GLS) can detect early subclinical LV dysfunction. The aim of this study was to evaluate the association of conventional echocardiographic parameters and GLS in relation to cardiovascular events in CHL survivors treated with thoracic radiotherapy. Impaired GLS was associated with increased risk of cardiovascular events. In addition, conventional echocardiographic parameters, including LVEF and diastolic dysfunction also showed a significant association with cardiovascular events and cardiac death. Assessing LV strain by echocardiography can contribute to early detection of subclinical LV dysfunction and identifying CHL patients at increased risk for cardiovascular events.

**Abstract:**

*Background:* Treatment with thoracic irradiation for classic Hodgkin lymphoma (CHL) leads to improved survival but also increases the risk of cardiovascular events. Left ventricular (LV) dysfunction is usually assessed by echocardiographic left ventricular ejection fraction (LVEF), whereas global longitudinal strain (GLS) can detect early subclinical LV dysfunction. The purpose of this study was to evaluate if conventional echocardiographic parameters and GLS are associated with cardiovascular events during long-term follow-up. *Methods:* 161 consecutive CHL patients treated with radiotherapy who underwent echocardiography > 10 years after diagnosis were assessed for eligibility. Multivariable cause-specific Cox regression was performed for a composite outcome of cardiac death and cardiovascular events and the competing outcome of noncardiac death. *Results:* 129 patients (61.2% female, *N* = 79) with a mean age of 46.3 ± 11.0 years at index visit were eligible for analysis. GLS was impaired in 51 patients (39.5%) and 10.9% had a LVEF of< 50%. The median E/e’ was 9.2 [7.2;12.7]. Adjusted for confounders, GLS > −16% showed a significant association with a near four-fold risk of the composite endpoint (HR = 3.95, 95% CI: 1.83–8.52, *p* < 0.001). LVEF < 50% (HR = 2.99, *p* = 0.016) and E/e’ (HR = 1.16, *p* < 0.001) also showed a significant relationship with the outcome. None of the aforementioned parameters were associated with the competing outcome. *Conclusions:* This study shows that LV dysfunction including impaired GLS in CHL survivors is associated with cardiovascular events and cardiac death.

## 1. Introduction

Long-term survival after classic Hodgkin lymphoma (CHL) has improved significantly in recent years, resulting from major improvements in (radiotherapeutic and chemotherapeutic) treatment possibilities [1,2], leading to a 10-year survival rate of> 80% [3,4,5]. As thoracic radiotherapy is one of the cornerstones of CHL treatment, improvement in its technique has contributed greatly to the survival of this patient group.

However, the long-term prognosis of this population is mainly determined by cardiovascular disease presenting as a late effect of radiotherapeutic treatment and the development of second malignancies [6,7,8,9,10]. The risk of cardiovascular disease is associated with the radiotherapeutic dose and location [11,12,13]. Patients treated with mediastinal radiotherapy are at risk of late cardiovascular problems, including accelerated development of atherosclerosis and valvular dysfunction [6,14,15,16,17]. Additionally, CHL patients treated with mantle field irradiation have shown relatively high incidence of (lethal) ischemic cardiac events [18]. Radiotherapeutic treatment in CHL patients is often combined with anthracyclines, which greatly contributes to the survival of CHL patients [2], but as exposure to anthracyclines—especially at a young age—is also known to increase the risk of cardiovascular disease [7,11,19,20]; this combination increases the risk of (late) cardiac events [8,13,21]. Therefore, cardiac death is the major nonmalignant cause of mortality in CHL survivors [4,22,23].

As CHL is mostly diagnosed at a young age, it is important to monitor cardiac function in these patients, and long-term follow-up including echocardiographic screening is recommended, preferably starting around 10 years after CHL diagnosis [11,24]. An increasing number of studies describe findings on echocardiographic changes after thoracic irradiation [25,26], including left ventricular (LV) diastolic and systolic dysfunction [25]. LV systolic function is usually assessed by left ventricular ejection fraction (LVEF) [27]. However, recent research has shown that measuring global longitudinal strain (GLS) can lead to earlier detection of subclinical LV dysfunction [27,28,29,30]. Nevertheless, the association between LV dysfunction and cardiac events after radiotherapy has not yet been investigated. Therefore, the purpose of this study was to evaluate if LV dysfunction is associated with an increased risk of cardiovascular events in CHL survivors treated with thoracic radiotherapy. This study focused on the value of conventional echocardiographic parameters of LV systolic and diastolic dysfunction as well as LV strain assessed by GLS in relation to the cardiovascular outcome.

## 2. Methods

### 2.1. Study Population

This single-center retrospective cohort study was conducted on patients treated with radiotherapy for CHL. Patients that were included in this study were referred to the outpatient cardio-oncology clinic at the Leiden University Medical Center as part of the national CHL survivorship care pathway. All CHL patients underwent a transthoracic echocardiogram at baseline visit. Patients were eligible for this study if they had a baseline visit at the outpatient clinic between 2003 and 2019 and had been treated for CHL with radiotherapy, alone or in combination with chemotherapy. The period of time between thoracic irradiation and baseline echocardiogram had to be at least 10 years, as the purpose of the study was to evaluate post-irradiation changes in echocardiographic parameters. Hypercholesterolemia was defined as a history of hypercholesterolemia or receiving cholesterol-lowering medication. Renal insufficiency was defined as an estimated glomerular filtration rate (eGFR) < 60 mL/min/1.73 m^2^. Furthermore, patients with a history of acute coronary syndrome (ACS) or coronary artery bypass grafting (CABG) were excluded as prior cardiovascular events could interfere with the association of echocardiographic abnormalities on future cardiac events. Lastly, patients with missing data on exposure to thoracic irradiation and/or chemotherapy were ineligible for this study. All patients gave informed consent for participation in this study. The study was approved by the local Medical Ethical Committee (METC-LDD G20.045) and complies with the Declaration of Helsinki.

### 2.2. Data Collection

Demographic patient data, laboratory results, medication, and traditional cardiovascular risk factors were collected through the departmental Cardiology Information System (EPD-Vision^®^) and pharmacy records. Information on oncological characteristics, including the date of CHL diagnosis, detailed information on radiotherapeutic and chemotherapeutic treatment (chemotherapy regimen, cumulative anthracycline dose, radiation dose, and fields of radiation), and Ann Arbor stage were collected from the internal oncology registry (OncDoc), which is connected to the Netherlands Cancer Registry. The cause of death was collected through the civil municipal registry. Cardiac events during follow-up were assessed through telephone calls, billing codes, and electronic health records.

### 2.3. Echocardiography

Echocardiographic images, using the Vivid 7, E9, and E95 systems (General Electric Healthcare Vingmed, Horten, Norway), were acquired with patients in the left lateral decubitus position. Data acquisition was performed using 3.5 MHz or M5S transducers. In addition to standard 2D and color images, continuous wave and pulsed wave Doppler images were obtained using dedicated software (Echopac BT13; GE Medical Systems, Milwaukee, WI, USA). The images were stored for offline analysis.

From the apical two- and four-chamber view, the LV end-diastolic and LV end-systolic volume indexes (LVEDVi, LVESVi) were assessed, and the LVEF was calculated using the modified Simpson rule [31]. The biplane Simpson method was used to calculate left atrial volume index (LAVi). Diastolic dysfunction was assessed by Doppler peak early (E-wave) and late (A-wave) velocities, deceleration time, and the E/A ratio. In addition, using Tissue Doppler imaging, the E/e’ ratio was calculated and peak early diastolic tissue velocity from the lateral and septal mitral annulus (e’-wave) was measured. Recommendations of the European Association of Cardiovascular Imaging (EACVI) and the American Society of Echocardiography (ASE) were implemented to grade diastolic function [32]. LV GLS was assessed using two-dimensional speckle-tracking strain analysis (EchoPAC version 203). This software was used to analyze deformation of the myocardium, which was assessed by tracking the movement of acoustic markers frame-to-frame. The LV endocardial border was traced in apical two-, three- and four-chamber views, and regions of interest could be adjusted manually. LV GLS was calculated automatically and defined as the average of the longitudinal strain values of the different apical views. Shortening of the LV myocardium is reflected by GLS as negative value. Therefore, a better deformation is defined by a more negative LV GLS value. Systolic dysfunction was defined as an LV GLS > −16% or LVEF < 50% [33,34]. According to most recommendations by the ASE/EACVI, diastolic dysfunction was identified by at least two of the following parameters: LAVi > 34 mL/m^2^, E/e’ ratio > 14, lateral e’ < 10 cm/s, septal e’ < 7 cm/s, and peak tricuspid regurgitation (TR) velocity > 28 m/s [32].

### 2.4. Study Endpoint

The primary endpoint of this study consisted of a composite endpoint of ACS, cardiac surgery, admission for heart failure (HF), and cardiac death. The competing outcome is defined as noncardiac death due to malignancy or other causes of death.

### 2.5. Statistical Analysis

Continuous variables are expressed as mean ± standard deviation or median (interquartile range (IQR)), depending on the normality of distribution, which was assessed graphically or with the Kolmogorov–Smirnov test. Categorical data is presented as frequencies or percentages. To determine the association of various echocardiographic parameters with the study endpoints in the presence of competing risk of noncardiac death, cause-specific Cox proportional hazard modeling was performed. Censoring took place at occurrence of the composite outcome (or competing outcome), lost to follow-up, or on the last known date of follow-up. Median follow-up time was estimated through the reverse Kaplan–Meier method. Both crude and adjusted hazard ratios (HR) for the composite study endpoint and competing endpoint are presented. The models were adjusted for the possible confounders age at index visit, gender, and the presence of cardiovascular risk factors at baseline (hypertension, hypercholesterolemia, positive family history of cardiovascular disease, LDL cholesterol > 3 mmol/L or total cholesterol > 5 mmol/L). The proportional hazard assumption was graphically assessed through Schoenfeld residuals plot, and the assumption holds for all models. Cumulative incidence curves were constructed for GLS > −16% or ≤−16% on the composite endpoint. Harrell’s C index was calculated to assess model performance. The adjusted models for GLS and LVEF were compared using a likelihood ratio test. A *p*-value of< 0.05 was considered as statistically significant. Missing data was accounted using complete case analysis. All statistical analyses were performed in STATA version 17.0 (StataCorp 2021, College Station, TX, USA).

## 3. Results

### 3.1. Study Population

**Figure 1:**
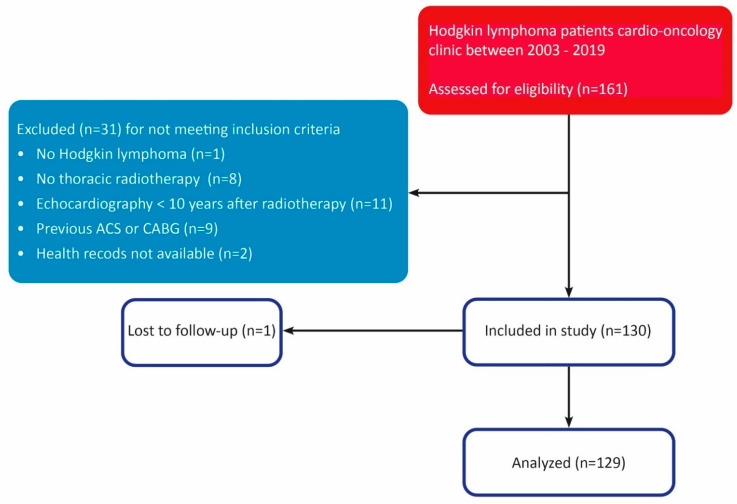
Strobe diagram. STROBE diagram of patient selection process. ACS = acute coronary syndrome, CABG = coronary artery bypass grafting.

In total, 161 patients were evaluated, 32 of which were excluded for analysis. The inclusion process is shown in the strobe diagram in Figure 1. After the selection process, 129 patients were eligible for analysis. The baseline characteristics of the study population are shown in Table 1. The majority of the study population consisted of women (*N* = 79, 61.2%). The median age of the study population at diagnosis was 24.4 [p25;p75: 18.8;29.0] years. Seventy-nine patients (61.2%) were treated additionally to radiotherapy with anthracyclines, with a median cumulative anthracycline dose of 210 [150;300] mg/m^2^. Fifty-four patients (41.9%) were treated with mediastinal irradiation, 46 patients (35.7%) with mantle field radiotherapy, 24 patients (18.6%) with subtotal radiotherapy, and 4 patients (3.1%) with another type of radiotherapy. The mean cumulative radiation dose was 36 [35;40] Gy. No significant differences in baseline characteristics were found between patients with the primary outcome versus patients without the primary outcome.

**Figure 2:**
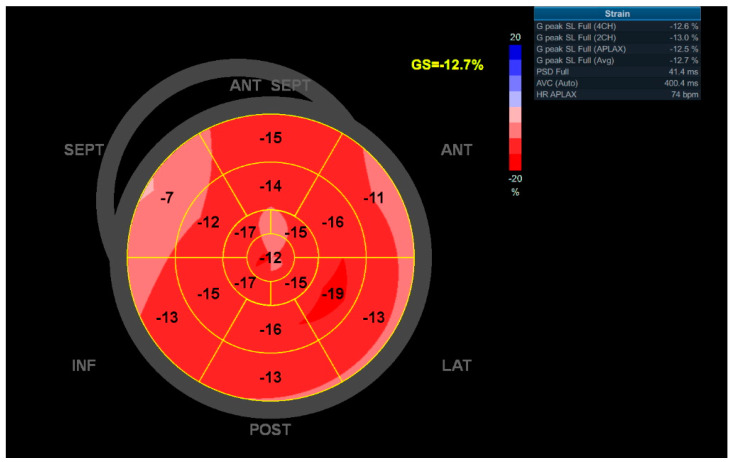
Figure 2 shows a Bulls-Eye longitudinal strain map of a 36-year-old woman who was diagnosed with CHL in 1999 and treated with chemotherapy and mantle field radiotherapy. Echocardiography at index visit in 2015 showed impaired LV GLS of −12.7% and LVEF of 48%. This patient had a grade 1 diastolic dysfunction and an E/e’ mean of 8.8. In 2017, this patient was admitted for HF due to non-ischemic cardiomyopathy, most probably caused by prior chemotherapy.

LV GLS could be determined in 98% of the patients. See Figure 2 for an example Bulls-Eye longitudinal strain map of a patient with impaired LV GLS. Median LV GLS was −−17.1% [−19;−15.2], and 51 patients (39.5%) had impaired systolic function defined by a LV GLS > −16%. The median LVEF at baseline was 55.0% [52.0;58.0], and 10.9% of the patients (*N* = 14) had a decreased LVEF < 50%. In 38 patients with a preserved ejection fraction, the LV GLS was declined. Regarding diastolic dysfunction at baseline, 49 patients (38.0%) had mild diastolic dysfunction (grade 1), 24 patients (18.6%) had moderate diastolic dysfunction (grade 2), and 8 patients (6.2%) had severe diastolic dysfunction (grade 3). All echocardiographic baseline characteristics of the study population are shown in Table 2. Diastolic dysfunction, LVEF, GLS, and resting heart rate (RHR) during echocardiography showed significant difference between the two groups with and without cardiovascular events.

### 3.2. Cardiovascular Outcome

The median follow-up period of this study cohort was 8.1 [4.6;9.6] years. For the patients who experienced the composite endpoint, median time to event since index visit was 1.7 [0.3;4.9] years and median time since radiotherapy was 28.1 [20.8;31.9] years. In total, the composite endpoint occurred 47 times: 3 patients (2.3%) presented with ACS, 27 patients (20.9%) underwent cardiac surgery (CABG: *N* = 5, CABG + valve surgery: *N* = 5, valve surgery: *N* = 11, transcatheter aortic valve implantation (TAVI): *N* = 4, mitraclip: *N* = 1, and ostiumplasty of the left main (LM) coronary artery: *N* = 1), and 7 patients (5.4%) were admitted for HF. Events are listed in Table 3. Survival status could be assessed for 129 patients (100%), and in all patients the cause of death was known. In total, 25 patients (19.4%) died during follow-up: 10 patients (7.8%) due to a cardiac cause, 12 patients (9.3%) due to a malignant cause, and 3 patients (2.3%) due to another cause of death. The overall 5-year survival from index visit of our study cohort was 89.1%, and the 10-year survival was 79.2%.

### 3.3. Echocardiographic Parameters Associated to the Cardiovascular Outcome

**Figure 3:**
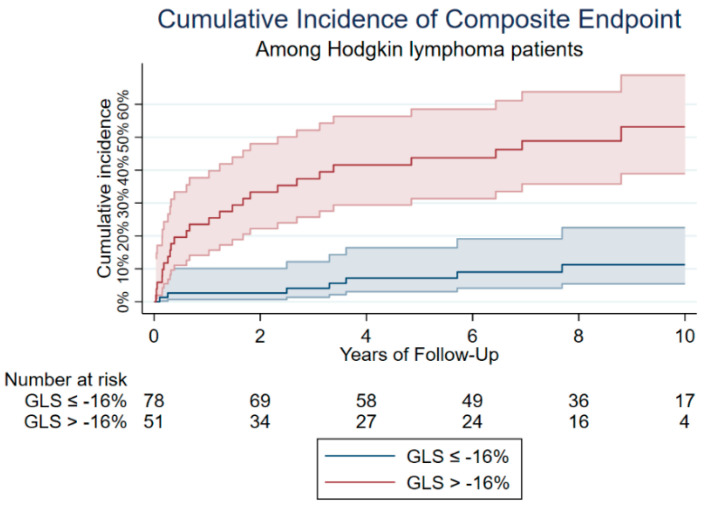
Cumulative incidence of composite endpoint. Cumulative incidence of the composite endpoint is shown for GLS ≤ −16% versus GLS > −16%. This figure shows that impaired GLS is associated with higher cumulative incidence of the composite endpoint.

Univariable Cox regression was performed followed by multivariable Cox regression adjusted for gender, age at index visit, and the presence of the predefined cardiovascular risk factors at baseline. Patients with a LVEF < 50% had a three-fold increased risk of cardiovascular events and cardiac death (HR_adj_ = 2.98, 95% CI = 1.22–7.29, *p* = 0.016). Diastolic dysfunction, assessed by E/e’ (HR_adj_ = 1.16, 95% CI = 1.09–1.22, *p* = <0.001) and a grade > 0 (HR_adj_ = 3.63, 95% CI = 1.04–12.70, *p* = 0.044) also showed a relationship with the primary outcome. Finally, LV GLS > −16% was significantly associated with an increased risk of the composite outcome (HR_adj_ = 3.95, 95% CI = 1.83–8.52, *p* = <0.001. A cumulative incidence curve for the composite endpoint was constructed for GLS > −16% versus GLS ≤ −16% (Figure 3).

**Figure 4:**
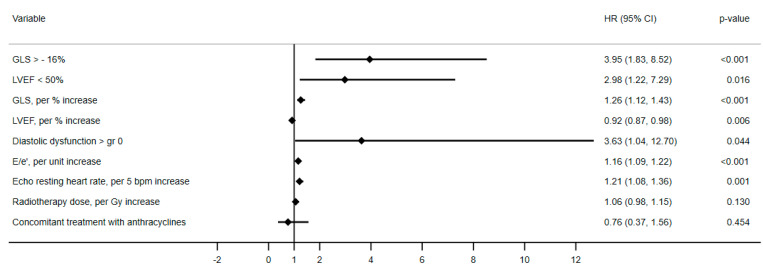
Forest plot of hazards ratios for the study outcome. Hazard ratios adjusted for gender, age, and cardiovascular risk factors at index visit are shown for the risk of the study outcome. None of the variables showed significant association with competing risk of noncardiac death.

Competing risk Cox regression for the competing outcome non-cardiovascular death was performed for all above mentioned variables, and none showed association with the competing risk. HRs are shown in Table 4 and the forest plot in Figure 4.

### 3.4. Performance of GLS Compared to LVEF to Predict Cardiac Events at Follow-Up

The Harrell’s C-statistic was calculated for the incremental value of GLS compared to LVEF in a model adjusted for gender, age at index visit, and cardiovascular risk factors at baseline. Although the C-statistic differed for GLS and LVEF (0.81 (95% CI: 0.74–0.88) and 0.77 (95% CI: 0.69–0.85) respectively), no significant difference in discriminating value between these two variables was found.

## 4. Discussion

This retrospective study shows that LV dysfunction assessed by conventional systolic and diastolic parameters as well as LV strain is frequently detected in CHL survivors treated with radiotherapy. In addition, these parameters were associated with worse cardiovascular outcome during long-term follow-up.

### 4.1. Echocardiographic Follow-Up in Cancer Patients

According to the most recent guidelines by the European Society of Cardiology (ESC) on cardio-oncology [24], echocardiography is the main imaging method of choice to detect myocardial dysfunction. It is recommended to estimate LVEF routinely using 3D-echocardiography or—if not available—2D-echocardiography using the biplane Simpson method before, during, and after cancer treatment for early detection of cardiac dysfunction. Although LVEF is the most commonly used parameter to assess LV function in relation to cancer treatment, research has shown the incremental value of GLS to detect subclinical LV dysfunction [27]. In the general population, impaired GLS is usually defined by a cut-off value of >−16% [33]. However, in studies focusing on the cardio-oncology population, various GLS cut-off values—ranging from −13.8% to −20.5%—are represented as a threshold for the detection of cancer-therapy-related cardiac dysfunction [35,36,37,38,39,40].

The ASE/EACVI expert consensus on multimodality imaging in cancer patients concluded that GLS is superlative compared to LVEF for early detection of subclinical LV dysfunction in cancer patients before, during, and after treatment [41]. However, despite all advantages and prognostic value of GLS, it requires specific expertise on echocardiographic techniques, and routine use in clinical practice has not been implemented in all centers [24,27]. Therefore, standardized acquisition protocols are being developed for routine implementation of echocardiographic strain measurement.

The recently published expert consensus by the international cardio-oncology society focusing on cardiovascular manifestations post-irradiation share several recommendations for cardiac monitoring after thoracic irradiation [42]. This consensus statement advises to perform a baseline thoracic echocardiogram and to monitor echocardiographic function every 5 years after thoracic irradiation. Patients were defined as high risk for cardiovascular disease if they had received mediastinal radiotherapy ≥ 30 Gy or mediastinal radiotherapy < 30 Gy in combination with anthracycline treatment, or if their age was below 50 years. The median age at diagnosis of our study population was 24.4 years, the median radiotherapeutic dose was 36.0 Gy, and 61.2% of the patients were concomitantly treated with anthracyclines. The current study supports the need for regular follow-up, as recommended by the consensus paper.

### 4.2. LV Dysfunction in CHL Survivors

Impairment in GLS can be observed earlier, before LVEF impairment occurs [2,43,44]. Only a few studies have assessed LV function including LV GLS in CHL survivors treated with radiotherapy. Tsai et al. found reduced strain in a small study of 47 CHL survivors compared to a healthy population [45]. In addition, the authors assessed GLS in patients all treated with radiotherapy and compared two groups with and without anthracycline chemotherapy. GLS was reduced in patients receiving anthracyclines with mediastinal radiotherapy compared to the other group receiving mediastinal radiotherapy alone. In addition, a very recent study by Van der Velde et al. confirmed LV changes in long-term CHL survivors with cardiac magnetic resonance imaging. The authors assessed differences in LVEF and strain in CHL patients treated with radiotherapy alone or in combination with chemotherapy versus non-CHL patients and also showed that strain parameters and LVEF were significantly worse in the CHL population [30]. Studies performed on breast cancer patients treated with radiotherapy alone confirmed that radiation can influence LV function, with a significant decline in GLS promptly after radiotherapeutic treatment as well as up to three years thereafter [46,47,48].

### 4.3. LV GLS in Relation to Cardiovascular Outcome

Several studies have shown the predictive value of echocardiographic strain on future LVEF decrease and cardiotoxicity in cancer patients treated with trastuzumab or anthracyclines [49,50]. Its incremental value to LVEF on predicting cardiovascular outcomes has been shown in several populations, including cardiac mortality in patients with chronic kidney disease, dilated cardiomyopathy (CMP), or acute HF and early post-operative outcomes after cardiac surgery [51,52,53,54]. In addition, a study by Rhea et al. found that strain was significantly associated with all-cause mortality in patients with cancer and a normal LVEF. Strain provided incremental prognostic value to clinical parameters [55]. To the best of our knowledge, this is the first study that has assessed the value of LV GLS in relation to cardiovascular events in CHL survivors treated with radiotherapy. Our study showed that both systolic and diastolic function parameters are related to long-term cardiovascular events. In line with the literature, LV dysfunction was more frequently detected by GLS in comparison to LVEF. However, no significant differences were observed in discriminating value between GLS and LVEF, which could be explained by the small sample size and relatively low number of events. Although this study does not have enough power to assess the incremental value of strain to LVEF, our results suggest that measuring both echocardiographic parameters can lead to earlier and improved identification of CHL patients with increased risk of cardiotoxicity. Nevertheless, it should be considered to start HF medication in patients with impaired strain, as this reflects subclinical LV dysfunction and may improve cardiovascular outcome.

### 4.4. Clinical Implications of LV Dysfunction in CHL Survivors

The current study shows that LV dysfunction detected during cardiac monitoring after radiotherapy in CHL survivors is associated with cardiovascular events during long-term follow-up. Current position statements do not recommend interference with cancer treatment as a result of (significantly) decreased GLS [27]. Therefore, risk stratification and consequent preventive cardiovascular treatment is considered as the most important value of assessing GLS. However, only a small number of studies have evaluated the effect of medication in cardiotoxicity, and the impact on clinical outcome is unclear. Following from the results of a study by Negishi et al., initiating beta blocker treatment in cancer patients treated with trastuzumab or anthracyclines based on impaired echocardiographic GLS can contribute to recovery of early cardiac dysfunction [56]. A study by Silber et al. that focused on treatment with enalapril in childhood cancer survivors found a decrease in left ventricular end-systolic wall stress, but treatment with enalapril did not lead to significantly improved outcomes [57]. Although another study showed that enalapril had a beneficial effect on the cardiac parameters of LV dysfunction—including LV afterload, LV mass, and LV dimension—in childhood cancer survivors, this effect only persisted for a maximum of 6 to 10 years [58]. Nevertheless, a study performed in cancer patients treated with chemotherapy has shown that treatment with the ACE-converting enzyme inhibitor enalapril in patients with an increased cardiac troponin I is useful to prevent echocardiographic cardiotoxicity [59]. This study defined cardiotoxicity by a decrease of > 10% in LVEF in combination with a drop in LVEF below 50%. However, a large multicenter randomized trial performed by Cardinale et al. showed no differences in troponin rise, and therefore no increased risk of LV dysfunction based on elevated troponin I as a predictor, between patients preventively treated with enalapril and patients treated with enalapril after the first troponin increase was measured [60]. Therefore, it could be speculated that initiating treatment with beta blockers or enalapril in HL survivors leads to reduced HF and improved outcomes.

Finally, (modifiable) lifestyle factors have been associated with worse prognosis in CHL survivors. Especially hypertension was found to be associated with increased risk of cardiovascular events [61]. As treatment with mediastinal radiotherapy increases the risk of cardiovascular events, patients should be encouraged to healthy lifestyles—including smoking cessation and moderate-intensity physical activity—to reduce the risk of future events [62].

### 4.5. Sex Differences

Our study cohort shows a predominance of females (61.2%). Studies have shown that, in general, cardiovascular disease develops at a younger age in men than in women. In addition, men are at higher risk for coronary artery disease, even in the absence of cardiovascular risk factors at baseline. Additionally, women tend to have a lower risk factor burden than men. Therefore, occurrence of cardiovascular events in our study population could be affected by sex-differences. However, all multivariable analyses were adjusted for gender.

### 4.6. Study Limitations

This is a retrospective single-center cohort study with a sample size of 129 patients and limited number of events. Due to this limited number of events and small sample size per radiotherapeutic field, it was not possible to analyze the separate hazard ratios for type of radiotherapeutic treatment and the composite endpoint. Additionally, routinely obtaining cardiovascular biomarkers of HF, including atrial natriuretic peptide and brain natriuretic peptide, has only been protocolized recently. Therefore, the association between levels of these biomarkers and impaired GLS could not be explored. Moreover, for the purpose of the study outcome, patients with previous ischemic heart disease (ACS, CABG) were excluded. Therefore, the implication of these results cannot be extrapolated to all CHL survivors.

## 5. Conclusions

LV dysfunction assessed by conventional echocardiographic parameters, as well as LV GLS, is frequently detected in CHL survivors treated with radiotherapy. LVEF, diastolic dysfunction, and LV GLS were significantly associated with cardiac events and cardiac death. With LV GLS, subclinical LV dysfunction can be detected in an early stage, identifying patients at risk for adverse events.

## Figures and Tables

**Table 1:** Baseline characteristics of the study population.

	Total (*N* = 129)	No Events (*N* = 94)	Events (*N* = 35)	*p*-Value
Female	79 (61.2%)	65 (69.1%)	14 (40.0%)	0.003
BMI, kg/m^2^	24.8 ± 4.8	24.5 ± 4.4	25.5 ± 5.7	0.31
Age at diagnosis, yrs	24.4 [18.8;29.0]	23.1 [18.7;28.2]	26.0 [20.2;30.5]	0.19
Cardiovascular risk factors				
Hypertension	26 (20.2%)	15 (16.0%)	11 (31.4%)	0.051
Diabetes Mellitus	4 (3.1%)	2 (2.1%)	2 (5.7%)	0.30
Hypercholesterolemia	20 (15.5%)	12 (12.8%)	8 (22.9%)	0.16
Positive family history of cardiovascular disease	25 (19.4%)	19 (20.2%)	6 (17.1%)	0.69
Smoking	6 (4.7%)	3 (3.2%)	3 (8.6%)	0.20
Congestive heart failure	1 (0.8%)	0 (0.0%)	1 (2.9%)	0.100
Ischemic heart disease	0 (0.0%)	0 (0.0%)	0 (0.0%)	
Renal insufficiency	4 (3.1%)	3 (3.2%)	1 (2.9%)	0.92
Laboratory results				
Hb, mmol/L	8.7 ± 0.9	8.5 ± 0.8	9.0 ± 0.9	0.006
Leukocytes, × 10^9^/L	7.4 ± 2.9	7.0 ± 1.8	8.4 ± 4.6	0.019
LDL, mmol/l	3.1 ± 1.0	2.9 ± 0.8	3.8 ± 1.2	<0.001
Total cholesterol, mmol/L	5.3 ± 1.2	5.1 ± 1.1	5.8 ± 1.3	0.007
Creatinine, µmol/L	73.1 ± 15.0	71.1 ± 14.9	78.9 ± 13.8	0.012
Stage Hodgkin Lymphoma				0.67
I–II	96 (74.4%)	69 (73.4%)	27 (77.1%)	
III–IV	33 (25.6%)	25 (26.6%)	8 (22.9%)	
Location of radiotherapy				0.032
Mantle field	46 (35.7%)	27 (28.7%)	19 (54.3%)	
Mediastinal	54 (41.9%)	46 (48.9%)	8 (22.9%)	
Subtotal	24 (18.6%)	17 (18.1%)	7 (20.0%)	
Other	5 (3.9%)	4 (4.3%)	1 (2.9%)	
Radiotherapeutic dose, Gy	36.0 (35.0;40.0)	36.0 (35.0;40.0)	36.0 (35.0;40.0)	0.47
Treated with chemotherapy	101 (78.3%)	74 (78.7%)	27 (77.1%)	0.85
Chemotherapeutic regimen (*N* = 101)				0.51
ABVD	29 (22.5%)	21 (22.3%)	8 (22.9%)	
MOPP/ABV	28 (21.7%)	22 (23.4%)	6 (17.1%)	
EBVP	9 (7.0%)	7 (7.4%)	2 (5.7%)	
BEAUCOPP	5 (3.9%)	5 (5.3%)	0 (0.0%)	
MOPP	20 (15.5%)	12 (12.8%)	8 (22.9%)	
Other	9 (7.0%)	6 (6.4%)	3 (8.6%)	
Treated with anthracyclines	79 (61.2%)	61 (64.9%)	18 (51.4%)	0.16
Type of anthracycline (*N* = 79)				0.87
Doxorubicine	71 (55.0%)	55 (58.5%)	16 (45.7%)	
Epirubicine	8 (6.2%)	6 (6.4%)	2 (5.7%)	
Cumulative dose anthracycline, mg/m^2^	210.0 [150.0;300.0]	210.0 [140.0;280.0]	210.0 [200.0;300.0]	0.36

Laboratory reference values: Hb 7.5–10.0 mmol/L (f)/8.5–11 mmol/L (m), leukocytes 4.00–10.00 × 10^9^/L, LDL < 3 mmol/L, total cholesterol < 5 mmol/L, creatinine 49–90 (f)/64–104 (m). BMI = body mass index; Hb = hemoglobin; LDL = low-density lipoprotein; ABVD = Adriamycin, Bleomycin, Vinblastine, Dacarbazine; MOPP = Mustargen, Vincristine, Procarbazine, Prednisone; ABV = Adriamycin, Bleomycin, Vinblastine; EBVP = Epirubicin, Bleomycin, Vinblastine, Prednisone; BEAUCOPP = Bleomycin, Etoposide, Adriamycin, Cyclophosphamide, Vincristine, Procarbazine, Prednisone; MOPP = Mustargen, Vincristin, Procarbazine, Prednisone.

**Table 2:** Echocardiographic characteristics.

	Total (*N* = 129)	No Events (*N* = 94)	Events (*N* = 35)	*p*-Value
Body surface area during echocardiogram	1.9 [1.7;2.0]	1.9 [1.7;2.0]	2.0 [1.8;2.1]	0.026
LVESV, mL	38.0 [31.0;47.0]	36.0 [28.0;46.0]	42.0 [35.0;56.0]	0.014
LVESVi, mL/m^2^	20.1 [16.2;25.0]	19.6 [15.5;24.7]	22.0 [17.3;26.9]	0.054
LVEDV, mL	83.0 [68.0;102.0]	80.0 [64.0;97.0]	93.0 [74.0;111.0]	0.044
LVEDVi, mL/m^2^	45.9 [36.0;52.6]	44.3 [35.6;51.2]	46.1 [36.6;56.3]	0.17
Mitral regurgitation				0.54
None	90 (69.8%)	68 (72.3%)	22 (62.9%)	
Mild	25 (19.4%)	18 (19.1%)	7 (20.0%)	
Moderate	12 (9.3%)	7 (7.4%)	5 (14.3%)	
Severe	2 (1.6%)	1 (1.1%)	1 (2.9%)	
E, m/s	77.0 [66.0;89.0]	76.5 [67.0;88.0]	79.0 [59.0;102.0]	0.75
A, m/s	75.0 [59.0;93.0]	71.0 [55.0;90.0]	83.5 [69.0;110.0]	0.026
Deceleration time, ms	196.0 [168.0;251.0]	194.0 [168.0;245.0]	200.0 [167.0;277.0]	0.42
e’ mean, cm/s	8.0 [6.5;10.0]	9.0 [7.0;10.5]	6.5 [5.0;7.5]	<0.001
E/e’ mean	9.1 [7.2;12.7]	8.7 [6.8;11.1]	13.6 [8.6;18.9]	<0.001
Diastolic dysfunction				<0.001
Grade 0	46 (35.7%)	43 (45.7%)	3 (8.6%)	
Grade 1	49 (38.0%)	31 (33.0%)	18 (51.4%)	
Grade 2	24 (18.6%)	16 (17.0%)	8 (22.9%)	
Grade 3	8 (6.2%)	3 (3.2%)	5 (14.3%)	
Diastolic dysfunction > grade 0	81 (63.8%)	50 (53.8%)	31 (91.2%)	<0.001
LAVi, mL/m^2^	18.4 [15.5;22.8]	18.8 [16.1;22.8]	16.6 [13.9;22.9]	0.38
LVEF, %	55.0 [52.0;58.0]	56.0 [53.0;59.0]	53.0 [50.0;56.0]	0.004
LVEF < 50%	14 (10.9%)	7 (7.4%)	7 (20.0%)	0.042
GLS, %	−17.1 [−19.0;−15.2]	−17.5 [−19.3;−15.9]	−14.9 [−16.2;−13.3]	<0.001
GLS > −16%	51 [39.2%]	26 [27.4%]	25 [71.4%]	<0.001
RHR during echocardiography	77.5 [70.0;88.5]	76.0 [70.0;86.0]	85.0 [74.0;94.0]	0.009

LVESV = left ventricular end-systolic volume, LVESVi = indexed left ventricular end-systolic volume, LVEDV = left ventricular end-diastolic volume, LVEDVi = indexed left ventricular end-diastolic volume, E = E wave, A = A wave, e’ mean = the average of e’ septal and e’ lateral, LAVi = left atrial volume index, LVEF = left ventricular ejection fraction, GLS = global longitudinal strain, RHR = resting heart rate.

**Table 3:** Cardiovascular events during median follow-up time of 8.1 years.

Cardiovascular Events	*N* = 129
ACS	3 (2.3%)
Cardiac surgery	27 (20.9%)
Type of cardiac surgery	
CABG	5 (3.9%)
CABG + Valve surgery	5 (3.9%)
Valve surgery	11 (8.5%)
TAVI	4 (3.1%)
Mitraclip	1 (0.8%)
Ostiumplasty of LM coronary artery	1 (0.8%)
Admission for HF	7 (5.4%)

Percentages of events are shown as cumulative incidence proportions of the total study population. ACS = acute coronary syndrome, CABG = coronary artery bypass grafting, TAVI = transcatheter aortic valve implantation, LM = left main, HF = heart failure.

**Table 4:** Univariable and Multivariable hazard ratios for the composite endpoint.

	Univariable		Multivariable	
Variable	HR (95% CI)	*p*-Value	HR (95% CI)	*p*-Value
GLS > −16%	4.92 (2.36–10.27)	<0.001	3.95 (1.83–8.52)	<0.001
LVEF < 50%	2.32 (1.01–5.40)	0.048	2.98 (1.22–7.29)	0.016
E/e’, per 1 unit increase	1.16 (1.10–1.21)	<0.001	1.16 (1.09–1.22)	<0.001
GLS, per % increase	1.29 (1.16–1.44)	<0.001	1.26 (1.12–1.43)	<0.001
LVEF, per % increase	0.93 (0.88–0.99)	0.014	0.92 (0.87–0.98)	0.006
Diastolic dysfunction > gr 0	6.01 (1.84–19.68)	0.003	3.63 (1.04–12.70)	0.044
Echo RHR, per 5 bpm increase	1.15 (1.03–1.29)	0.015	1.21 (1.08–1.36)	0.001
Radiotherapy dose, per Gy increase	1.07 (0.98–1.16)	0.112	1.06 (0.98–1.15)	0.130
Concomitant treatment with anthracyclines	0.60 (0.30–1.17)	0.132	0.76 (0.37–1.56)	0.454

Hazard ratios are shown with 95% confidence intervals and *p*-values for univariable as well as multivariable Cox regression adjusted for gender, age at index visit, and predefined risk factors at baseline. GLS = global longitudinal strain, LVEF = left ventricular ejection fraction, RHR = resting heart rate, Gy = gray.

## Data Availability

The data presented in this study are available on request from the corresponding author. The data are not publicly available duo to privacy reasons.
